# Impact of radiation dose on neurocognitive function and quality of life in long-term survivors of childhood brain tumour

**DOI:** 10.2340/1651-226X.2025.43989

**Published:** 2025-09-18

**Authors:** Laura Toussaint, Anne Sophie Lind Helligsø, Ludvig Paul Muren, Ali Amidi, Rikke Hedegaard Dahlrot, Louise Tram Henriksen, Maja Vestmø Maraldo, Martin Skovmos Nielsen, Anouk Kirsten Trip, Lisa Maria Wu, Yasmin Lassen-Ramshad

**Affiliations:** a Danish Centre for Particle Therapy, Aarhus University Hospital, Aarhus, Denmark; b Department of Clinical Medicine, Aarhus University, Aarhus, Denmark; c Department of Pediatrics and Adolescent Medicine, Aarhus University Hospital, Aarhus, Denmark; d Department of Pediatrics and Adolescent Medicine, Gødstrup Hospital, Gødstrup, Denmark; e Unit for Psycho Oncology & Health Psychology, Department of Psychology and Behavioral Sciences, Aarhus University, Aarhus, Denmark; f Department of Oncology, Odense University Hospital, Odense, Denmark; g Department of Clinical Research, University of Southern Denmark, Denmark; h Department of Oncology, Copenhagen University Hospital – Rigshospitalet, Copenhagen, Denmark; i Department of Clinical Medicine, University of Copenhagen, Copenhagen, Denmark; j Department of Oncology, Aalborg University Hospital and Clinical Cancer Research Center, Aalborg, Denmark; k Department of Psychology, Reykjavik University, Reykjavik, Iceland

**Keywords:** Paediatrics, central nervous system tumours, quality of life, neurocognition, late effects, radiotherapy

## Abstract

**Background and purpose:**

Children treated with radiotherapy (RT) for a brain tumour often exhibit neurocognitive impairment and report lower quality of life (QoL) later in life. The aim of this nationwide cross-sectional cohort study was to explore the impact of RT dose to brain organs at risk (OARs) on neurocognition and QoL in long-term survivors of childhood brain tumours.

**Patient/material and methods:**

A total of 132 survivors of childhood brain tumours, diagnosed from 2001 to 2017 in Denmark, underwent neurocognitive tests and QoL questionnaires at least 5-years post-diagnosis. Neurocognitive assessments were complete and available for 86 patients (61 no-RT/25 RT), and QoL scores for 107 (79 no-RT/28 RT). Mann Whitney U-tests were used to compare scores between no-RT and RT groups. For scores impacted by RT, OAR-specific robust linear regressions were performed to evaluate RT dose effects while adjusting for potential confounders.

**Results:**

Clinically significant overall cognitive impairment was observed for 55% of the neurocognitive sub-cohort, with younger age at treatment time as a significant risk factor, while hydrocephalus status had no impact. There were no statistically significant differences on neurocognitive tests between the RT and no-RT group. However, patients treated with RT had significantly lower scores on the physical and social functioning QoL domains, with mean dose to the pituitary gland and left hippocampus, respectively, as significant predictors.

**Interpretation:**

This cross-sectional study indicates that RT dose-effects, particularly in the pituitary gland and left hippocampus, might contribute to reduced QoL in survivors of childhood brain tumours.

## Introduction

In recent years, survival rates of paediatric brain tumour patients have increased [1], in part due to progress in the multimodality management of the disease, earlier detection, and improved follow-up supportive care. However, survivors of central nervous system (CNS) tumours experience the highest cumulative burden of chronic health conditions compared to both the general population and survivors of other paediatric cancers [2]. In particular, survivors of CNS tumours suffer from neurocognitive impairments in a broad range of domains, including attention, working memory, and processing speed [3]. In turn, these can impact education, future employment, or quality of life (QoL) in a broader sense [4].

A study from the St Jude Lifetime cohort reported that survivors of paediatric CNS tumours, with a median of 18 years follow-up, were at higher risk of neurocognitive impairment in adult life than the general population, and whole brain irradiation was associated with the highest risks [5]. Although based on a much shorter time since diagnosis (median of 2.9 years), a prospective longitudinal study of proton-irradiated patients recently reached similar conclusions, with patients treated with surgery-only or focal proton therapy having stable neurocognitive scores over time, comparable to normative values, while patients treated with whole brain irradiation were at risk for, for example, processing speed impairment [6].

For patients treated with radiotherapy (RT), a recent review from the Pediatric Normal Tissue Effects in Clinic (PENTEC) group linked brain RT dose/volume to intelligence quotient (IQ) scores [7]. Higher RT doses to brain substructures have previously been associated with neurocognitive impairment in survivors of a paediatric brain tumour. Higher mean doses to the cerebellum have been correlated with lower full-scale IQ and lower performance on processing speed, working memory, and perceptual reasoning [8], while higher hippocampus doses have been associated with impairment in delayed memory [9].

A Danish nationwide cohort study of long-term survivors of paediatric brain tumours showed that 66% of all survivors exhibited overall neurocognitive impairment (i.e. clinical impairment in at least two cognitive domains) at a mean time of 15 years since diagnosis. Hydrocephalus and younger age at treatment were predictors of overall neurocognitive impairment. Patients treated with RT, especially when receiving whole brain irradiation, exhibited lower neurocognitive scores than patients not receiving RT. The highest impairments were found for processing speed and sustained attention. Similar trends were seen for global QoL as well as social and physical functioning [10]. However, no detailed RT dose parameters for specific brain organs at risk (OARs) were included in the analysis.

The aim of the present study was therefore to further explore the potential impact of delivered RT dose to specific OARs on neurocognitive function and QoL in a subset of this nationwide Danish cohort of long-term survivors of childhood brain tumours.

## Patients/material and methods

### Cohort description and treatment

**Figure 1:**
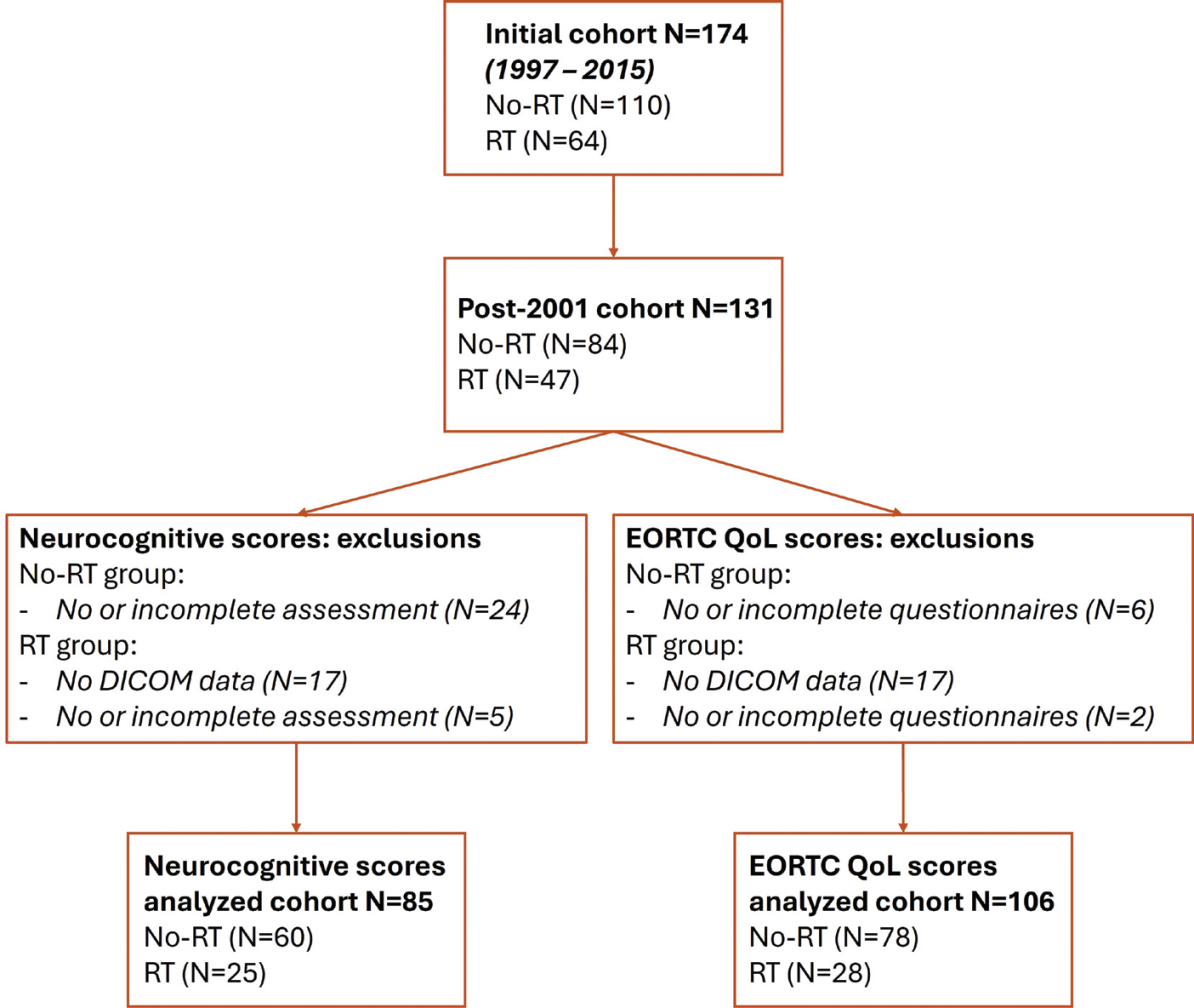
Flow chart of the patients included in both analyses. The initial cohort is described in [10]. Briefly, inclusion criteria were: confirmed diagnosis of a brain tumour at the age 15 years or younger, more than 5 years since diagnosis, and at least 15 years old at the time of clinical examination. Exclusion criteria were evidence of disease progression and intraspinal tumour. RT: radiotherapy; DICOM: digital imaging and communications in medicine; EORTC: European Organization for Research and Treatment of Cancer; QoL: quality of life.

From the initial 174 patients in the established Danish cohort [10], 132 childhood survivors of brain tumours diagnosed from 2001–2015 were considered for inclusion in this study. Year 2001 was chosen as a threshold date to ensure RT treatment plans could be retrieved in Digital Imaging and Communications in Medicine (DICOM) format from the treatment planning systems. Patients were identified through the Danish Childhood Cancer Registry, and underwent neurocognitive assessments and QoL questionnaires at least 5-years post-diagnosis (in the period August 2019–September 2021) [10]. Applying the exclusion criteria as outlined in Figure 1, neurocognitive scores of 86 patients (61 no-RT/25 RT) and QoL scores of 107 patients (79 no-RT/28 RT) were available for analyses.

Clinical follow-up evaluations were performed at a median of 12 years post-treatment (minimum 5 years–maximum 20 years in both sub-cohorts). In both the neurocognitive and QoL sub-cohorts, patients’ characteristics were overall similar in the no-RT and RT-group. Only use of chemotherapy differed, with 64% (61% for the QoL sub-cohort) of the patients in the RT group receiving chemotherapy as part of their treatment, compared to 7% in the no-RT group (5% for the QoL sub-cohort). A detailed summary of demographic and clinical details is available in Table 1.

**Table 1:** Summary of the patient characteristics for the two analyses.

	Neurocognitive tests		EORTC QoL scores	
	No-RT (*N* = 60)	RT (*N* = 25)	No-RT (*N* = 78)	RT (*N* = 28)
**Female**	32 (53%)	17 (68%)	42 (54%)	17 (61%)
**Age at treatment**	10.2 years	9.5 years	9.4 years	10.1 years
*median (IQR)*	(6.6–13.3)	(6.5–11.9)	(5.9–13.0)	(7.3–12.1)
**Time since diagnosis**	12.4 years	12.4 years	12.6 years	12.4 years
*median (IQR)*	(8.8–16)	(8.5–14.6)	(9.1–16.9)	(9.2–14.6)
**Whole brain irradiation**	*NA*	10 (40%)	*NA*	12 (43%)
**Surgery**	53 (88%)	22 (88%)	67 (86%)	24 (86%)
**Chemotherapy**	4 (7%)	16 (64%)	4 (5%)	17 (61%)
**Hydrocephalus**	16 (27%)	7 (28%)	18 (23%)	9 (32%)

RT: radiotherapy; IQR: interquartile range; EORTC: European Organization for Research and Treatment of Cancer; QoL: Quality of Life.

In both sub-cohorts, the median prescription dose in the RT group was 54 Gy (minimum 24 Gy–maximum 59.4 Gy/68 Gy in the neurocognitive/QoL sub-cohorts, respectively). Of the 25 RT-treated patients in the neurocognitive sub-cohort, 10 received whole-brain irradiation, of whom 2 with protons. Seven patients treated with focal RT also received protons. Of the 28 RT-treated patients in the QoL sub-cohort, 12 received whole-brain irradiation, of whom 2 with protons. Seven patients treated with focal RT also received protons.

For all patients treated with RT, the individual treatment plans were collected from the treating institution, and included computed tomography (CT) scan, structure set, and dose maps in DICOM format. OAR not delineated in the structure set of the clinical plans were retrospectively contoured. The analysed OARs and dose metrics included mean dose (Dmean) to the whole brain, supratentorial brain, cerebellum, brainstem, pituitary gland, hippocampus (left/right) and temporal lobe (left/right) [8], as well as the volume of hippocampus (left/right) receiving 40 Gy (V40Gy) [9] and the volume of brain receiving 30 Gy (V30Gy).

### Test battery

The neurocognitive test battery was chosen based on recommendations from the International Cognition and Cancer Task Force [11], testing the following six domains: processing speed (Trail Making Test A, Coding subtest of the Wechsler Adult Intelligence Scale version IV [WAIS-IV]), sustained attention (detectability, omission and commission scores from the Conners’ Continuous Performance Test version III), attention and working memory (Digit Span subtest of the WAIS-IV), verbal learning and memory (total, delayed and retention scores from the Hopkins Verbal Learning Test-Revised), verbal fluency (letter S and animals subtests of the Controlled Oral Word Association Test), and executive functions (Trail Making Test B). An age-adjusted z-score was calculated based on normative data. An age-adjusted z-score of 0 shows no difference between the tested cohort and normative values while a negative z-score represents an impairment in the tested cohort compared to normative performance, with a z-score ≤ −1.5 representing clinically significant impairment on a specific neurocognitive test. Patients were categorised as having overall clinically significant cognitive impairment when they had impairment (z-score ≤ −1.5) in at least two different cognitive domains [11]. QoL was assessed using the European Organization for Research and Treatment of Cancer (EORTC) core questionnaire (EORTC QLQ-C30) for global QoL and five functional scales (physical, role, emotional, cognitive, and social functioning) [12]. Scores ranged from 0 to 100, with lower scores reflecting lower performance in the tested function. Sex- and age-specific normative data from the Danish population were available for reference [13]. A decrease of 10 points or more compared to normative values was chosen as the clinically significant threshold, corresponding to a small-to-medium minimally important difference on the QLQ-C30. The detailed test battery is summarised in Supplementary Table 1, and all tests were administered in the Danish language.

### Hypotheses and statistical analysis

This study was based on three hypotheses: for all neurocognitive tests and QoL scores, patients treated with RT will have lower scores than patients who did not receive RT; patients receiving higher radiation dose to specific OARs will perform worse on neurocognitive tests and QoL scores; and patients treated at a younger age or experiencing hydrocephalus will have lower neurocognitive scores.

As normal distribution of the data could not be assumed (one-sample Kolmogorov-Smirnov test), median values and interquartile range (IQR) were reported. Two-sided Mann Whitney U-tests were used to compare neurocognitive and QoL outcomes between groups, and Chi-Square tests for categorical variables. A *p-*value ≤ 0.05 was considered statistically significant.

If significant differences were identified for a given outcome, OAR-specific linear regression models with backward selection were performed to evaluate RT dose effects while adjusting for potential confounders, including age at treatment, time since diagnosis, sex, whole-brain irradiation, chemotherapy, surgery, and hydrocephalus. One model per OAR dose metric (mentioned above) was developed. If several models were statistically significant, the most predictive one was selected based on comparison of R-squared and adjusted R-squared values, as well as *p*-value. Multicollinearity was also assessed using variation inflation factors (VIFs). The final model was then refined using robust linear regression with bootstrapping (1,000 samples) to estimate the 95% confidence intervals (CIs), reducing reliance on normality assumptions.

## Results

For both the neurocognitive and QoL sub-cohorts, no differences were observed in the RT dose/volume metrics between the focal and whole-brain irradiation groups for the brainstem, pituitary gland, and hippocampi. The largest dose differences between the focal RT and whole-brain irradiation groups were seen for the cerebellum Dmean, with a median of 24.1 Gy versus 53.6 Gy, respectively (23.8 Gy vs. 53.6 Gy in the QoL cohort). Radiotherapy dose/volume metrics to OARs are summarised in Supplementary Table 2.

Clinically significant overall cognitive impairment was observed for 55% of the neurocognitive sub-cohort (47/85 patients). Age at treatment was significantly different between patients with and without clinically significant cognitive impairment (*p* < 0.01), with patients treated at a younger age presenting more clinically significant cognitive impairment, while no difference was seen in terms of time since diagnosis (*p* = 0.6). There was also no statistically significant difference in terms of hydrocephalus between the two groups (Chi-Square test, *p* = 0.53) (Table 2).

**Table 2:** Summary of the patient characteristics for patients without overall clinically significant cognitive impairment and those with.

	Overall Neurocognitive Impairment	
	No (*N* = 38, 45%)	Yes (*N* = 47, 55%)
**Female**	22 (58%)	27 (57%)
**Age at treatment**	12.1 years	9 years
*median (IQR)*	(7.3–14.3)	(5.5–11.5)
**Time since diagnosis**	12.7 years	12.4 years
*median (IQR)*	(8.6–16.9)	(8.9–15.2)
**Radiotherapy**	9 (24%)	16 (34%)
**Whole brain irradiation**	3 (8%)	7 (15%)
**Surgery**	33 (87%)	42 (89%)
**Chemotherapy**	7 (18%)	13 (28%)
**Hydrocephalus**	9 (24%)	14 (30%)

IQR: interquartile range.

**Figure 2:**
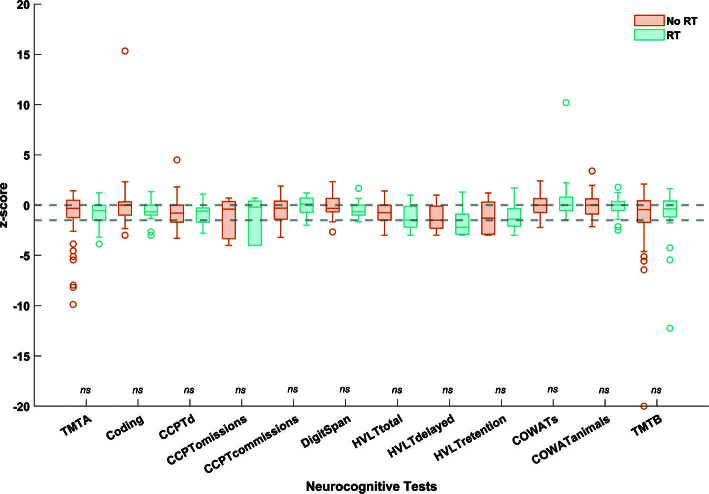
Boxplots of the z-scores from all neurocognitive tests in the cohort treated without (orange) versus with (blue) radiotherapy. The dotted lines represent the z-score thresholds for normative mean value (z-score = 0) and clinically significant impairment (z-score ≤ −1.5). ns: non-significant (Mann Whitney U-test); TMT: Trail Making Test; CCPT: Conners’ Continuous Performance Test; HVLT-R: Hopkins Verbal Learning Test-Revised; COWAT: Controlled Oral Word Association Test.

When comparing the neurocognitive scores to the normative mean scores in both the no-RT and RT groups, the median z-score was lower than the normative mean z-score for almost all tests. Clinically significant impairment (i.e. z-score ≤ −1.5) in verbal learning and memory was observed in one test in the no-RT group (Hopkins Verbal Learning Test-Revised [HVLT-R] delayed test, z-score = −1.5), and two tests in the RT group (HVLT-R delayed and total tests, z-score = −1.5 and −2.2, respectively). When comparing the no-RT to the RT group, no statistically significant differences were observed on any of the individual neurocognitive test scores (Figure 2, Supplementary Table 3).

**Figure 3:**
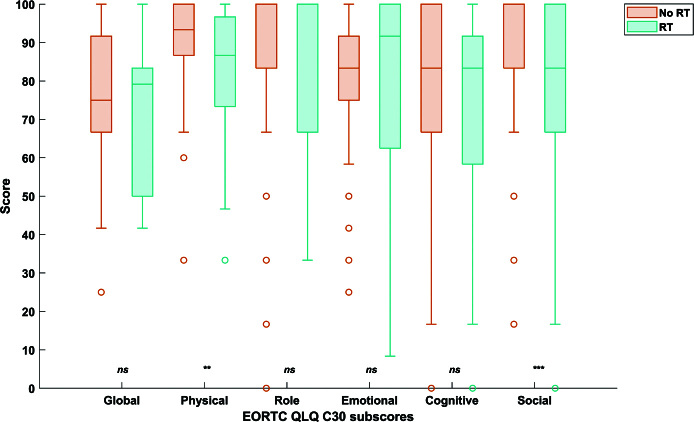
Boxplots of the scores from the EORTC QLQ C30 questionnaire in the cohort treated without (orange) versus with (blue) radiotherapy. For all items, a higher score represents better functioning. ns: non-significant, ** p < 0.01, *** p < 0.001 in the Mann Whitney U-test.

When comparing QoL scores between the no-RT and the RT group, RT-treated patients had significantly lower scores in physical and social functioning (*p* < 0.01). For physical functioning, the median (IQR) score was 93.3 [86.7–100.0] for the no-RT versus 86.7 [73.3–96.6] for the RT group. For social functioning, the median (IQR) score was 100.0 [83.3–100.0] for the no-RT versus 83.3 [66.7–100.0] for the RT group (Figure 3, Supplementary Table 4).

**Figure 4:**
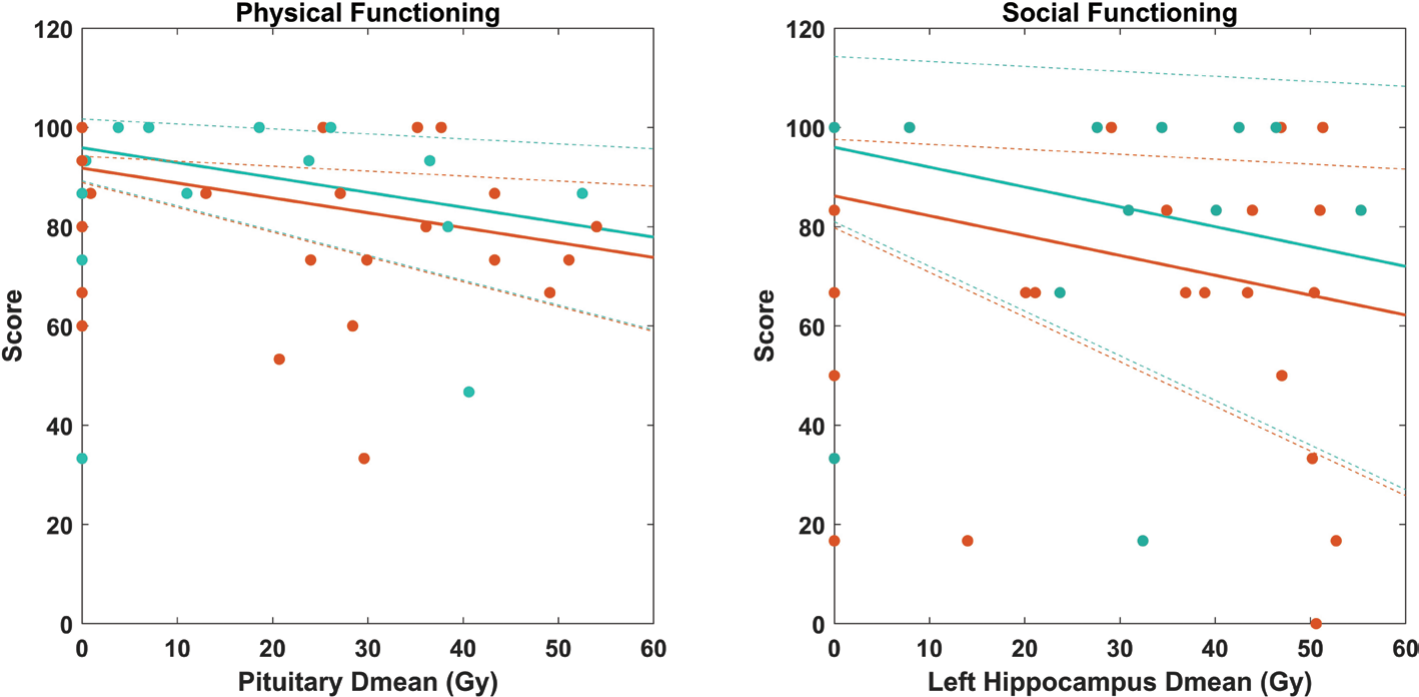
Representation of the robust linear regression models (full line), with 95% confidence interval (dotted lines) for physical functioning (left panel) and social functioning (right panel) scores. Each dot represents one patient of the analysed QoL cohort. RT dose metrics for patients in the no-RT group were set to 0. Models for male patients are represented in cyan, and the model for female patients in orange. For clarity of the figure, the two models for social functioning scores are shown for ‘no chemotherapy use’ only.

All OAR dose metrics but the hippocampus left V40Gy were statistically significant predictors of physical functioning scores in the backward selection process. The model based on the mean dose to the pituitary gland (ranging from 9 Gy to 38 Gy in the RT-group) was the strongest statistically significant predictor of physical functioning scores, and also included sex as a variable. For social functioning scores, all OAR dose metrics but the Dmean to the cerebellum and the brain V30Gy were statistically significant predictors of social functioning scores in the backward selection process. The model based on the left hippocampus mean dose (ranging from 25.1 Gy to 48.3 Gy in the RT-group) was the most statistically significant, and also included sex and chemotherapy as variables. In both cases, a higher mean dose resulted in lower QoL sub-scores. For both models, no problematic multicollinearity was detected with all predictors having a VIF ≤ 1.55 (Supplementary Table 5). The two final predictive robust linear regressions are summarised in Table 3 and Figure 4.

**Table 3:** Results of the robust linear regressions with bootstrapping estimates of the 95% confidence interval for each parameter.

	Value	95% CI	SE	*t*-value
*Physical functioning*				
**Intercept**	91.8	(88.9, 94.2)	1.24	74.2
**Pituitary gland**Dmean (Gy)	−0.3	(−0.5, −0.1)	0.06	−5.3
**Male = 1** **Female = 0**	4.1	(0.3, 7.5)	1.69	2.4
*Social functioning*				
**Intercept**	86.2	(79.8, 97.6)	2.64	32.7
**Left hippocampus** Dmean (Gy)	−0.4	(−0.9, −0.1)	0.12	−3.5
**Male = 1** **Female = 0**	9.8	(1.2, 16.7)	3.55	2.9
**Chemotherapy** (Yes = 1/No = 0)	7.4	(−4.9, 22.9)	5.48	1.4

Physical functioning score = intercept + a x dose + b x sex, social functioning score = intercept + a x dose + b x sex + c x chemotherapy.

CI: confidence interval; SE: standard error; Dmean: mean dose.

## Discussion and conclusion

The present study explored the potential impact of delivered RT dose to specific OARs on neurocognitive function and QoL in a nationwide cohort of long-term survivors of childhood brain tumours. Patients presenting with clinically significant overall neurocognitive impairment were younger than those without clinically significant impairment at the time of treatment, but no difference was seen in terms of hydrocephalus status between those groups. While no difference in neurocognitive function was observed between patients treated with and without RT, the study indicated that higher RT doses to particularly the pituitary gland and left hippocampus may contribute to reduced physical and social functioning, respectively, in survivors of childhood brain tumours.

The association between higher mean RT dose delivered to the pituitary gland and lower physical functioning seems to be in alignment with a recent report from the PENTEC group showing that RT dose to the hypothalamic-pituitary axis was associated with the risk of growth hormone deficiency [14]. In turn, common symptoms of growth hormone deficiency include, for example, decreased muscle mass and function or decreased mineral bone density [15], potentially influencing physical function (i.e. the ability to perform everyday activities). Of note, management of hormone deficiencies after RT has been an integral part of clinical routine in Denmark. Even though this management is of relevance, it was out of the scope of this study to evaluate its potential impact on QoL. Normative values from Danish 20–29 years old reported a mean (standard deviation) of 97 (6) and 95 (11) for male and female, respectively [13], suggesting that the physical functioning scores in the RT group (median 86.7) is close to a clinically relevant impairment (i.e. ≥ 10 points decrease to normative values).

Similarly, a higher mean dose delivered to the left hippocampus and female sex were predictive parameters for lower social functioning scores. While no difference was seen in individual neurocognitive test scores between the RT and no-RT groups, we still observed clinically significant impairment (i.e. z-score ≤ −1.5) in verbal learning and memory in both groups. It could be hypothesised that left hippocampus integrity plays a key role in social functioning, for example through verbal learning and memory tasks. Impairments in this function could result in poorer social functioning through loss of memory of specific events. The median (IQR) social functioning score in the RT group was 83.3 [66.7–100.0], significantly lower compared to the no-RT group (100.0 [83.3–100.0]). Normative values from Danish 20–29 years old reported a mean (standard deviation) of 98 (8) and 95 (15) for male and female, respectively [13], suggesting that the impairment to social functioning in the RT group could be interpreted as clinically significant (i.e. ≥ 10 points decrease to normative values). The proposed robust linear regression seems to be in alignment with the direction of this relationship, although at a larger magnitude, with male sex resulting in a 9.8 (1.3, 15.9) points higher score than female sex on social functioning. Although impairment in neurocognitive scores could hypothetically also influence social functioning, this connection was not investigated in this study as no correlations between neurocognitive test results and QoL scores were reported in the initial full cohort we based our hypotheses upon [10].

In another study of QoL in survivors of childhood brain tumour, albeit based on a different questionnaire (i.e. PedsQL v4.0), social and physical functioning were also rated worse by survivors than by healthy children. Survivors scored 70% in social functioning compared to 95% for healthy controls, and 75% compared to 88% in physical functioning [16]. A recent review showed that, overall, social and physical functioning was scored lower than comparisons especially for female patients and those with a brain tumour diagnosis [17].

The mean dose to the pituitary gland and the left hippocampus were significant predictors for worse physical and social functioning, respectively. While not a statistically significant predictor, chemotherapy exposure was kept through the backward selection process as a protective factor, and therefore reported in the proposed social functioning model. Overall, the proposed models should not be assumed to be reliable for absolute estimates of QoL sub-scores, as seen with a comparably large spread of scores among patients with 0 Gy versus with RT dose in both models. Instead, it is of relevance to assess which parameters matter to the scores when adjusting for potential confounders. Those results, if confirmed in other cohorts, could guide RT dose planning, with, for example, further efforts towards reducing the mean dose to the pituitary gland or left hippocampus when possible. However, several dose parameters to other OARs were also predictive of QoL sub-scores (although to a lesser extent). One should therefore carefully assess potential undesirable RT dose redistribution when sparing specific OARs, and more data is needed to implement a holistic informed dose sparing strategy.

The low overall numbers in our study prevented us from investigating the potential impact of RT modality on the QoL scores. Specifically, only 9/28 patients from the QoL cohort were treated with proton therapy. Yock et al. reported that patients treated with proton therapy scored higher on QoL scores than patients treated with photon therapy, with physical functioning scores in proton-treated patients even comparable to healthy populations [18]. In general, proton plans result in lower doses delivered to the whole brain and OARs for various tumour sites [19]. However, a recent systematic review concluded that there was insufficient evidence to confirm that proton therapy could reduce the risk of impaired QoL compared to photon-based RT, and therefore advocated for a general effort toward implementing clinically standardised QoL assessment in this patient population in order to gain new insights [20].

Our results did not show a difference in neurocognitive scores between the group treated without versus with RT, which stands in contrast to the original Danish cohort where patients treated with RT (and especially those receiving whole brain irradiation) performed worse in, for example, processing speed and sustained attention tests compared to the no-RT patients [10]. Other studies have also highlighted greater risks amongst survivors who receive craniospinal irradiation (CSI) [5–6]. However, in the present sub-cohort analysis, such results could not be confirmed, which may be explained by the exclusion of patients treated in earlier years. Compared to the original cohort, 50 no-RT (26 of whom were treated before 2001) and 39 RT patients (17 of whom were treated before 2001) were excluded from the present analysis. Therefore, patients treated with less conformal plans and potentially receiving a higher dose bath to normal brain tissues were not included in the present analysis. Over the past decades, there has been a large evolution in RT technology and quality [21], with one study even suggesting that modern RT techniques affect neurocognitive function less than in the past [22]. The lack of effect in the present analysis may also be explained by the heterogeneity in RT modality (photon vs. proton). Finally, the cohort was also of limited size and largely imbalanced, with more patients in the no-RT versus RT group, potentially limiting our ability to detect a difference in neurocognitive impairment between groups, and to detect the impact of RT dose on potential neurocognitive side effects. These factors could also potentially explain the different findings in this area compared with the initial full cohort [10].

Some limitations of this study include that it was based on a relatively small cohort of patients with heterogeneous diagnoses. The participation rate was also relatively low (40.2% in the original cohort [10]), which could potentially introduce bias, with survivors with more clinically significant impairment perhaps less likely to agree to participate. At the same time, it is also important to highlight that patients treated with RT had inherently more aggressive tumours than patients in the no-RT group, and the impact of the tumour itself could also be contributing to observed QoL outcomes. Furthermore, more patients from the RT group received chemotherapy. The imbalance between the no-RT and RT group, with more patients not receiving RT, also reduced the statistical power to be able to detect an RT dose-effect. There was also no baseline QoL score or neurocognitive evaluation available for this cohort, making it difficult to determine the impact of the disease on these outcomes compared to the impact of the delivered treatment.

A strength of this study is that it examined a cohort of survivors drawn from a high-quality national registry, who were diagnosed based on the same national protocol and across the same time period. Moreover, survivors were tested with a comprehensive test battery at a median of 12 years post-treatment, facilitating the assessment of long-term effects.

To conclude, in this heterogeneous cohort of limited size, no difference in neurocognitive function was seen between patients treated with versus without RT, while physical and social functioning appeared to be lower for patients treated with RT. While younger age at diagnosis was associated with clinically significant neurocognitive impairment, the presence of hydrocephalus was not. These findings indicate that RT dose effects, particularly in the pituitary gland and left hippocampus, may contribute to reduced QoL in survivors of childhood brain tumours. Further clinical studies are warranted to confirm these findings.

## Supplementary Material



## Data Availability

To protect the privacy of research participants, the data are not publicly available. Data supporting the findings of this study are available from the corresponding author upon reasonable request.

## References

[1] Helligsoe ASL, Kenborg L, Henriksen LT, Udupi A, Hasle H, Winther JF. Incidence and survival of childhood central nervous system tumours in Denmark, 1997–2019. Cancer Med. 2022;11(1):245–56. 10.1002/cam4.4429 34800006 PMC8704152

[2] Bhakta N, Liu Q, Baassiri M, Eissa H, Yeo F, Chemaitilly W, et al. The cumulative burden of surviving childhood cancer: an initial report from the St. Jude Lifetime cohort study. Lancet. 2017;390:2569–82. 10.1016/S0140-6736(17)31610-0 28890157 PMC5798235

[3] Pancaldi A, Pugliese M, Migliozzi C, Blom J, Cellini M, Iughetti L. Neuropsychological outcomes of children treated for brain tumours. Children. 2023;10:472. 10.3390/children10030472 36980030 PMC10046931

[4] Ellenberg L, Liu Q, Gioia G, Yasui Y, Packer RJ, Mertens A, et al. Neurocognitive status in long-term survivors of childhood CNS malignancies: a report from the childhood cancer survivor study. Neuropsychology. 2009;23:705–17. 10.1037/a0016674 19899829 PMC2796110

[5] Brinkman TM, Krasin MJ, Liu W, Armstrong GT, Ojha RP, Sadighi ZS, et al. Long-term neurocognitive functioning and social attainment in adult survivors of pediatric CNS tumours: results from the St Jude Lifetime cohort study. J Clin Oncol. 2016;34:1358–67. 10.1200/JCO.2015.62.2589 26834063 PMC4933131

[6] Kahalley LS, Ris MD, Mahajan A, Okcu MF, Chintagumpala M, Paulino AC, et al. Prospective, longitudinal comparison of neurocognitive change in pediatric brain tumour patients treated with proton radiotherapy versus surgery only. Neuro Oncol. 2019;21:809–18. 10.1093/neuonc/noz041 30753584 PMC6558072

[7] Mahajan A, Stavinoha PL, Rongthong W, Brodin PN, McGovern SL, El Naqa I, et al. Neurocognitive effects and necrosis in childhood cancer survivors treated with radiation therapy: a PENTEC comprehensive review. Int J Radiat Oncol Biol Phys. 2024;119:401–16. 10.1016/j.ijrobp.2020.11.073. 33810950

[8] Söderström H, Walfridsson A, Martinsson U, Isacsson U, Brocki K, Kleberg JL, et al. Neurocognition and mean radiotherapy dose to vulnerable brain structures: new organs at risk? Radiat Oncol. 2023;18:132. 10.1186/s13014-023-02324-2 37568180 PMC10416465

[9] Acharya S, Wu S, Ashford JM, Tinkle CL, Lucas JT, Qaddoumi I, et al. Association between hippocampal dose and memory in survivors of childhood or adolescent low-grade glioma: a 10-year neurocognitive longitudinal study. Neuro Oncol. 2019;21:1175–83. 10.1093/neuonc/noz068 30977510 PMC7594551

[10] Helligsoe ASL, Henriksen LT, Kenborg L, Lassen-Ramshad Y, Wu LM, Winther JF, et al. Neurocognitive function and health-related quality of life in a nationwide cohort of long-term childhood brain tumour survivors. Neurooncol Pract. 2022;10:140–51. 10.1093/nop/npac085 36970169 PMC10037941

[11] Wefel JS, Vardy J, Ahles T, Schagen SB. International cognition and cancer task Force recommendations to harmonise studies of cognitive function in patients with cancer. Lancet Oncol. 2011;12:703–8. 10.1016/S1470-2045(10)70294-1 21354373

[12] Aaronson NK, Ahmedzai S, Bergman B, Bullinger M, Cull A, Duez NJ, et al. The European Organization for Research and Treatment of Cancer QLQ-C30: a quality-of-life instrument for use in international clinical trials in oncology. J Natl Cancer Inst. 1993;85:365–76. 10.1093/jnci/85.5.365 8433390

[13] Juul T, Petersen MA, Holzner B, Laurberg S, Christensen P, Grønvold M. Danish population-based reference data for the EORTC QCQ-C30: associations with gender, age and morbidity. Qual Life Res. 2014;23:2183–93. 10.1007/s11136-014-0675-y 24676897

[14] Wheeler G, Grassberger C, Samers J, Dwyer M, Wiltshire K, Daly P, et al. Central endocrine complications among childhood cancer survivors treated with radiation therapy: a PENTEC comprehensive review. Int J Radiat Oncol Biol Phys. 2024;119:457–66. 10.1016/j.ijrobp.2023.04.024 37269265

[15] Feldt-Rasmussen U, Klose M. Adult growth hormone deficiency: clinical management. In: Feingold KR, Anawalt B, Boyce A, editors. Endotext. South Dartmouth, MA: MDText.com, Inc; 2000. [Accessed May 29th 2025] Available from: https://www.ncbi.nlm.nih.gov/books/NBK425701/

[16] Musiol K, Bulska W, Brozek P, Oslizlo B, Ryzak S, Dubiel J, et al. Quality of life in survivors of childhood brain tumour and the association of children’s diseases on quality of their parents life. Psychooncology. 2019;28:1088–95. 10.1002/pon.5061 30875709

[17] Larsen PA, Amidi A, Ghith N, Winther JF, Pedersen C. Quality of life of adolescent and adult survivors of childhoodcancer in Europe – a systematic review. Int J Cancer. 2023;153:1356–75. 10.1002/ijc.34634 37377041

[18] Yock TI, Bhat S, Szymonifka J, Yeap BY, Delahaye J, Donaldson SS, et al. Quality of life outcomes in proton and photon treated pediatric brain tumour survivors. Radiother Oncol. 2014;113:89–94. 10.1016/j.radonc.2014.08.017 25304720 PMC4288853

[19] Toussaint L, Indelicato DJ, Stokkevåg CH, Lassen-Ramshad Y, Pedro C, Mikkelsen R, et al. Radiation doses to brain substructures associated with cognition in radiotherapy of pediatric brain tumours. Acta Oncol. 2019;58:1457–62. 10.1080/0284186X.2019.1629014. 31271084

[20] Doig M, Bezak E, Parange N, Gorayski P, Bedford V, Short M. Can we compare the health-related quality of life of childhood cancer survivors following photon and proton radiation therapy? A systematic review. Cancers. 2022;14:3937. 10.3390/cancers14163937 36010929 PMC9405962

[21] Ludmir EB, Grosshans DR, Woodhouse KD. Radiotherapy advances in pediatric neuro-oncology. Bioengineering. 2018;5:97. 10.3390/bioengineering5040097 30400370 PMC6315761

[22] Weusthof K, Lüttich P, Regnery S, König L, Bernhardt D, Witt O, et al. Neurocognitive outcomes in pediatric patients following brain irradiation. Cancers. 2021;13:3538. 10.3390/cancers13143538 34298751 PMC8307409

