# Supplementary material for: Impact of radiation dose on neurocognitive function and quality of life in long-term survivors of childhood brain tumour
Source: Acta Oncol. 2025 Sep 18;64:43989. doi: 10.2340/1651-226X.2025.43989 (PMC12452035; doi:10.2340/1651-226X.2025.43989)
Supplement: Supplementary file 1 [file AO-64-43989-s1.pdf]

Supplementary material has been published as submitted. It has not been copyedited, or typeset by Acta Oncologica

Supplementary Material

Supplementary Table 1: Neurocognitive tests and quality of life items tested in the study. Abbreviations: TMT = Trail Making Test, CCPT = Conners' Continuous Performance Test, HVLT-R = Hopkins Verbal Learning Test-Revised, COWAT = Controlled Oral Word Association Test, QoL = quality of life.

Domain	Test	Description
**Processing speed**	TMT-A	Drawing a line in sequence between numbers randomly displayed on a page.
	Coding	Copying symbols that correspond with numbers according to a key.
**Sustained attention**	CCPT detectability	14-minute trial. Responding when any letter appears, except the non-target letter "X". Inattentiveness, impulsiveness, sustained attention, and vigilance are assessed.
	CCPT omission	
	CCPT commission	
**Attention and working memory**	Digit span	Recalling strings of random digits presented aloud. Trials include forward, backward and ordered recall.
**Verbal learning and memory**	HVLT-R total	Recalling a list of 12 words presented over 3 trials. The total number of words recalled across 3 learning trials are combined into a total score, and a delayed recall trial after 20-25
	HVLT-R delayed	
	HVLT-R retention	

		minutes is used to generate a delayed recall and retention score.
**Verbal fluency**	COWAT letter S	Recalling as many words as possible beginning with the letter S during a 1-minute period.
	COWAT Animals	Naming as many animals as possible during a 1-minute period.
**Executive function**	TMT-B	Alternately connecting numbers and letters displayed on a page in numeric and alphabetical order.
**EORTC QLQ C30 questionnaires**	Global QoL	Global well-being (physical and psychological).
	Physical functioning	Ability to perform everyday activities (e.g. walking, standing…).
	Role functioning	Ability to perform daily work and activities.
	Emotional functioning	Level of emotional distress.
	Cognitive functioning	Ability to concentrate, memorize, think.
	Social functioning	Ability to have social interactions and relationships.

Supplementary Table 2: Median (IQR) dose metrics for OARs of interest for the two sub-cohorts. Dose metrics are reported for all RT patients, and for patients treated with focal vs. whole brain RT. The asterisk indicates a statistically significant difference between focal and whole brain RT patients, based on the Mann Whitney U-test. *Abbreviations: Dmean = mean dose, Brain sup = supratentorial brain, VxGy = volume of OAR receiving x Gy.*

		Neurocognitive tests			EORTC QoL scores		
		**All (N=25)**	**Focal (N=15)**	**Whole brain (N=10)**	**All (N=28)**	**Focal (N=16)**	**Whole brain (N=12)**
**Dmean (Gy)**	**Brain**	19.3 (13.3 – 33.7)	14.1* (10.1 – 18.6)	34* (31.5 – 35.6)	20 (13.6 – 34)	14* (10.3 – 18.2)	34.1* (31.1 – 35.9)
	**Brain Sup**	19.1 (10.4 – 30.8)	10.7* (8.5 – 16)	31.2* (27.9 – 32.4)	19.5 (10.7 – 31.2)	11.4* (9 – 15.7)	31.2* (28.6 – 34)
	**Cerebellum**	38.9 (20.2 – 53.2)	24.1* (5.3 – 38.9)	53.6* (46.8 - 54)	38.2 (23.1 – 53.5)	23.8* (7.4 – 38.5)	53.6* (42.8 – 54.9)
	**Brainstem**	*40.8* (32.2 – 50.6)	40.3 (30.9 – 49.7)	49.3 (40.3 – 52.2)	40.7 (33.1 – 50.8)	40.3 (32.1 – 49.5)	49.3 (40.2 – 52.5)
	**Pituitary gland**	28.4 (17.2 – 37.9)	29.6 (8 – 38.2)	27.7 (25.3 – 36.5)	29 (19.6 – 39.5)	25.2 (9 – 38)	32.3 (25.7 – 41.9)
	**Hippocampus L**	40.1 (26.6 – 47.8)	40.1 (20.3 – 45.8)	42.9 (34.4 – 50.6)	41.3 (30 – 50.3)	41.3 (25.1 – 48.3)	42.9 (33.4 – 50.8)
	**Hippocampus R**	41.9 (33.6 – 49.7)	41.9 (34.2 – 49.2)	41.4 (33.9 – 51.8)	43.3 (33.6 – 50.1)	43.3 (35.6 – 49.4)	41.4 (33.6 – 51.8)
	**Temporal Lobe L**	29.7 (19.9 – 38.4)	22.3* (9.8 – 33.1)	39.2* (30.7 – 42.1)	31.2 (21.2 – 38.7)	22.5* (11.5 – 33.7)	39.2* (31.2 – 42.3)
	**Temporal Lobe R**	31.9 (21.6 – 36.9)	22.9* (17.7 – 32.9)	37.8* (31.9 – 42.1)	32.2 (22.5 – 37.7)	24* (18.3 – 32.8)	37.9* (32.4 – 42.8)
**V40Gy (%)**	**Hippocampus L**	33.3 (3.2 – 97)	27.7 (1.4 – 80.1)	52.9 (4.1 – 97.4)	50 (4.9 – 98.1)	50.1 (7 – 89.9)	52.9 (2.3 – 98.7)

	**Hippocampus R**	69.5 (4.5 – 99.5)	69.5 (8.7 – 96.6)	60.5 (0 – 99.4)	73.2 (10.4 – 99.7)	73.3 (21.1 – 97.4)	60.6 (0 – 99.7)
**V30Gy (%)**	**Brain**	23 (14.3 – 38.5)	18.1* (12.1 – 22.8)	39.2* (26.7 – 44.7)	24.5 (14.9 – 41.1)	16.9* (12.3 – 22.6)	41.1* (31.3 – 71.9)

Supplementary Table 3: Median (IQR) z-scores of neurocognitive outcomes for survivors treated without vs. with radiotherapy. The p-values correspond to the Mann Whitney u-test, testing the hypothesis that the RT group will have lower scores than the no-RT group. Abbreviations: TMT = Trail Making Test, CCPT = Conners' Continuous Performance Test, HVLT-R = Hopkins Verbal Learning Test-Revised, COWAT = Controlled Oral Word Association Test, RT = radiotherapy.

Domain	Test	No-RT (N=60)	RT (N=25)	p-value
**Processing speed**	**TMT-A**	-0.3<br>(-1.2 – 0.5)	-0.5<br>(-1.5 – 0)	*0.58*
	**Coding**	0<br>(-1 – 0.3)	-0.7<br>(-1 – 0)	*0.17*
**Sustained attention**	**CCPT detectability**	-0.8<br>(-1.7 – 0)	-0.6<br>(-1.7 – -0.3)	*0.93*
	**CCPT omission**	-0.4<br>(-3.3 – 0.3)	-0.2<br>(-4 – 0.4)	*0.65*
	**CCPT commission**	-0.3<br>(-1.4 – 0.4)	0.1<br>(-0.7 – 0.7)	*0.29*
**Attention and working memory**	**Digit span**	-0.3<br>(-0.7 – 0.7)	-0.7<br>(-1 – 0)	*0.17*
**Verbal learning and memory**	**HVLT-R total**	-0.7<br>(-1.5 – 0)	-1.5<br>(-2.2 – -0.1)	*0.27*
	**HVLT-R delayed**	-1.5<br>(-2.3 – 0.1)	-2.2<br>(-2.9 – -0.9)	*0.22*
	**HVLT-R retention**	-1.3<br>(-2.9 – 0.3)	-1.4<br>(-2.1 – -0.3)	*0.95*
**Verbal fluency**	**COWAT letter S**	0<br>(-0.7 – 0.6)	0<br>(-0.6 – 0.8)	*0.35*
	**COWAT Animals**	0<br>(-0.9 – 0.6)	0<br>(-0.5 – 0.4)	*0.99*
**Executive function**	**TMT-B**	-0.4<br>(-1.7 – 0.4)	-0.4<br>(-1.2 – 0.4)	*0.68*

Supplementary Table 4: Median (IQR) QoL scores for survivors treated without vs. with radiotherapy. The p-values correspond to the Mann Whitney u-test, testing the hypothesis that the RT group will have lower scores than the no-RT group. The presented normative values are extracted from [10], as mean (SD) of 168 Danish responders in the age groups 20-29 years old (reflecting the analyzed cohort best). Abbreviations: RT = radiotherapy, QoL = quality of life, M = male, F = female.

	**No-RT (N=78)**	**RT (N=28)**	**p-value**	**Normative**
**Global QoL**	75<br>(66.7 – 91.7)	79.2<br>(50 – 83.3)	*0.13*	*M: 83 (15)*<br>*F: 78 (19)*
**Physical functioning**	93.3<br>(86.7 – 100)	86.7<br>(73.3 – 96.6)	*<0.01*<br>***	*M: 97 (6)*<br>*F: 95 (11)*
**Role functioning**	100<br>(83.3 – 100)	100<br>(66.7 – 100)	*0.32*	*M: 95 (11)*<br>*F: 90 (21)*
**Emotional functioning**	83.3<br>(75 – 91.7)	91.7<br>(62.5 – 100)	*0.79*	*M: 87 (15)*<br>*F: 83 (17)*
**Cognitive functioning**	83.3<br>(66.7 – 100)	83.3<br>(58.3 – 91.6)	*0.45*	*M: 93 (11)*<br>*F: 92 (14)*
**Social functioning**	100<br>(83.3 – 100)	83.3<br>(66.7 – 100)	*<0.001*<br>****	*M: 98 (8)*<br>*F: 95 (15)*

Supplementary Table 5: R-squared, adjusted R-squared, and p-value for the most statistically significant linear regression models of physical functioning and social functioning with backward selection. VIF values are also reported to assess multicollinearity of predictors. Abbreviations: Dmean = mean dose, Pit = pituitary, LeftHippo = left hippocampus, Chemo = chemotherapy, VIF = variation inflation factors.

**Backward-selected models**	**R-squared**	**Adjusted R-squared**	**p-value**	**VIF**
Physical functioning				
Intercept + a x DmeanPit + b x Sex	0.145	0.129	0.0003	Dmean: 1.016<br>Sex: 1.016
Social functioning				
Intercept + a x DmeanLeftHippo + b x Sex + c x Chemo	0.189	0.165	<0.0001	Dmean: 1.546<br>Sex: 1.007<br>Chemo: 1.541
